# Supplementary material for: MetaRibo-Seq measures translation in microbiomes
Source: Nat Commun. 2020 Jun 29;11:3268. doi: 10.1038/s41467-020-17081-z (PMC7324362; doi:10.1038/s41467-020-17081-z)
Supplement: Supplementary file 10 — Supplementary Data 7 [file 41467_2020_17081_MOESM10_ESM.zip › File2/Confidence_VeryHigh_Taxonomy/13111_out.krona.html]

Javascript must be enabled to view this page.

members
magnitude
magnitudeUnassigned
count
unassigned
taxon
rank

13111\_out

38

superkingdom
35
2

1239
35
phylum

186801
35
class

35
order
186802

2
family
31979

1485
1

SRS1041095\_contig\_number\_contig-100\_1570.1571
genus
2

1720194

SRS148511\_contig\_number\_14889
1
species

186803
family
33

11

SRS013158\_contig\_number\_contig-100\_12239.12239SRS014923\_contig\_number\_7789SRS015578\_contig\_number\_contig-100\_20356.113281SRS018817\_contig\_number\_contig-100\_24765.60005SRS023526\_contig\_number\_contig-100\_9495.9495SRS104084\_contig\_number\_contig-100\_33984.33985SRS142712\_contig\_number\_contig-100\_15620.15620SRS142890\_contig\_number\_contig-100\_16345.152724SRS143342\_contig\_number\_contig-100\_16196.16196SRS144183\_contig\_number\_contig-100\_40827.40828SRS147139\_contig\_number\_contig-100\_28644.61214
32
genus
189330

21
species
88431

SRS015217\_contig\_number\_26943SRS015431\_contig\_number\_475SRS015782\_contig\_number\_13202SRS016095\_contig\_number\_16152SRS017521\_contig\_number\_43870SRS019068\_contig\_number\_72440SRS049712\_contig\_number\_23446SRS049995\_contig\_number\_contig-100\_13109.93351SRS050422\_contig\_number\_28990SRS053356\_contig\_number\_contig-100\_285.262590SRS064276\_contig\_number\_37576SRS077392\_contig\_number\_15596SRS1041039\_contig\_number\_contig-100\_1180.119618SRS1041130\_contig\_number\_contig-100\_2183.2184SRS104636\_contig\_number\_8977SRS105153\_contig\_number\_contig-100\_14472.100166SRS143991\_contig\_number\_27512SRS148159\_contig\_number\_41726SRS148721\_contig\_number\_49258SRS149879\_contig\_number\_6419SRS893259\_contig\_number\_3990

1
genus
1213720

1
species
796942

SRS023604\_contig\_number\_4056

3

SRS043768\_contig\_number\_contig-100\_18104.69106SRS143598\_contig\_number\_13192SRS149879\_contig\_number\_contig-100\_14993.14994
